# *BRD4* and *PIN1* gene polymorphisms are associated with high pulse pressure risk in a southeastern Chinese population

**DOI:** 10.1186/s12872-020-01757-x

**Published:** 2020-11-04

**Authors:** Jin-jia Qiu, Rui-zhi Yang, Yi-jie Tang, Ying-yi Lin, Hao-jie Xu, Na Zhang, Min Liang, Hong-da Cai, Kai Zeng, Xiao-dan Wu

**Affiliations:** 1 Department of Anesthesiology, The First Affiliated Hospital, Fujian Medical University, Fuzhou, 350005 Fujian China; 2 Department of Anesthesiology, Fujian Provincial Hospital, Fujian Provincial Clinical Medical College, Fujian Medical University, Fuzhou, 350001 Fujian China

**Keywords:** High pulse pressure, *BRD4*, Single nucleotide polymorphisms

## Abstract

**Background:**

*BRD4* and *PIN1* have been described to be involved in inflammation and vascular endothelial cell dysfunction, which in turn may increase pulse pressure.

**Hypothesis:**

Genetic mutations within the *BRD4* and *PIN1* genes could affect the risk of high pulse pressure.

**Methods:**

A total of four single nucleotide polymorphisms (SNPs) (*BRD4*: rs4808278; *PIN1*: rs2233678, rs2287838, and rs2233682) were genotyped in a cohort of 666 hypertensive patients and 232 normotensive controls with Chinese Han origin. Generalized multifactor dimensionality reduction (GMDR) was used to screen the best interaction combination among the four SNPs within the *BRD4* and *PIN1* genes and diabetes. Logistic regression analysis was performed to calculate the odds ratio (ORs) (95% confidence interval (CI)) for the association between the four SNPs.

**Results:**

Adjusted for age, weight, waist circumference, drinking, smoking, hypertension, and diabetes, high pulse pressure risk was significantly higher for carriers with the rs4808278-TT genotype in *BRD4* than those with wild genotypes (OR: 0.400, 95% CI: 0.217–0.737, P* < 0.05). However, we did not find any significant association of rs2233678, rs2287838, and rs2233682 in *PIN1* with high pulse pressure susceptibility after covariate adjustment. GMDR analysis indicated a significant three-locus model (*P* = 0.0107) involving rs4808278, rs2233678, and diabetes, the cross-validation consistency of the three-locus models was 9/10, and the testing accuracy was 57.47%.

**Conclusions:**

Genetic mutations within *BRD4* (rs4808278) could affect the susceptibility to high pulse pressure in a southeastern Chinese population.

## Background

Pulse pressure (PP) refers to the difference between systolic and diastolic blood pressure, of which the normal range is 30–50 mmHg [1]. As an independent risk factor of cardiovascular disease, PP is attracting more attention from doctors, and its forecast value may be larger than systolic blood pressure (SBP) or diastolic blood pressure (DBP) [2]. Hypertension and cardiocerebrovascular diseases caused by vascular diseases are often accompanied by elevated PP [3–5]. Therefore, a comprehensive understanding of the molecular basis of high PP is crucial for the development of potential therapeutic strategies.

The decrease in vascular elasticity is an important factor for the increase in pulse differential pressure and may result from inflammation and vascular endothelial cell dysfunction. Many novel loci related to blood pressure (BP) adjustment have been identified by researchers. However, it is suggested that there is a possible presence of allelic heterogeneity in BP regulation between Europeans and Asians, which provide new mechanistic insights into cardiovascular disease [6]. A previous study has reported that *BRD4* and *PIN1* are involved in inflammation and vascular endothelial cell dysfunction. As a transcription regulatory factor, *BRD4* may lead to extracellular calcium deposition, and *BRD4* may cooperate with seven specific transcription factors to promote calcification [7]. *BRD4* silencing results in a significant reduction in inflammatory cytokine expression [8]. Multiple studies have shown that *PIN1* binds to endothelial nitric oxide (NO) synthase and enables dephosphorylation of serine, which increases NO production and endothelium-dependent dilation, leading to BP maintenance [9]. Inhibiting *Brd4* and *Pin1* expression alleviates enhancer-mediated inflammatory transcription and atherosclerosis, which indicates that *BRD4* and *PIN1* play a vital role in the development of vascular inflammation and atherosclerosis [10–13]. Therefore, it is possible that variants in *BRD4* and *PIN1* genes may lead to decreased vascular elasticity by destroying endothelial cells and causing atherosclerosis.

The aim of this study was to investigate the association between *BRD4* and *PIN1* gene polymorphisms and high PP in a Chinese population.

## Methods

### Subjects

Han Chinese who visited the Fujian Medical University Affiliated with the First Hospital (from September 2014 to December 2016) were potential study subjects for this research. Patients with renal failure, severe heart disease (e.g. myocardial infarction, congenital heart disease, serious arrhythmia, valvulopathy or other atherosclerotic lesions), gestation, severe psychiatric disorder, cancer, peripheral vascular disease, severe anemia, severe metabolic disease, or infectious disease were excluded. Therefore, 232 healthy control subjects (SBP < 140 mm Hg and DBP < 90 mm Hg) who were not taking antihypertensive medication and 666 hypertensive patients (SBP ≥ 140 mm Hg or DBP ≥ 90 mm Hg) were selected for the study. Also, BP was measured three times using a calibrated mercury sphygmomanometer by certified examiners with at least 10 min interval and taken the average of three readings. The difference between systolic and diastolic blood pressure is PP. The aims of the study were carefully explained to all participants, who signed an informed written consent. The project complied with the Declaration of Helsinki guidelines and was approved by the Institute's Human Research and Ethics Committees.

### Single nucleotide polymorphism (SNP) selection and genotyping

SNPs associated with risk factors for high pulse differential pressure and their minor allele frequencies are greater than 5% in the 1000 Genomes Project (https://www.internationalgenome.org/). We used DNA isolation kits (Taijing Biological Technology [Xiamen], China) to extract genomic DNA from blood samples collected in anticoagulant ethylene diamine tetraacetic acid(EDTA) tubes and stored at − 20 °C. Five SNPs (rs4808278, rs2233678, rs2233679, rs2287838, and rs2233682) were genotyped using the polymerase chain reaction ligase detection reaction (PCR-LDR) method with five TaqMan genotyping assays on a Light Cycler 480 Instrument (Roche Diagnostics, Basel, Switzerland). Taqman probes and primers for the *BRD4* and *PIN1* genes were synthesized by Shanghai Generay Biotech (see Additional file 2: Table S1, in the online version of this article). PCR amplification was carried out in a PTC-200 MJ Research Peltier Thermal Cycler (Bio-Rad Lab, Massachusetts, USA). Genotyping of the five examined SNPs was performed as previously described [14].

### Statistical analysis

SPSS v.25.0 was used for statistical analyses. Means and standard deviations (SDs) were calculated for normally distributed continuous variables, and percentages were calculated for categorical variables. The Student's t-test was used to compare normally distributed numeric variables between two groups, otherwise a non-parametric test was used. SHESIS software (https://www.nhgg.org/analysis/) was used to calculate Hardy–Weinberg equilibrium (HWE), genotype/allele frequencies, and haplotype analysis [15, 16]. To evaluate the association between SNPs and high PP, logistic regression analysis was used to examine the odds ratios (ORs) and 95% confidence intervals (CIs). Generalized multifactor dimensionality reduction (GMDR) was used to detect gene–gene and gene-environment interactions. *P* values reported were two-tailed and those less than 0.05 were considered statistically significant. The analysis results were also processed using an online calculator for false discovery rate (FDR) correction (https://www.sdmproject.com/utilities/?show=FDR).

## Results

### Clinical characteristics of the study population

We recorded baseline characteristics including anthropometric index and clinical biomarkers between patients with high (243 males, 217 females) and normal (275 males, 163 females) PP. As shown in Table 1, there was no statistical difference in weight (*P* > 0.05) between the two groups. In addition, cases and controls were comparable with respect to height, drinking, and smoking (*P* < 0.05). Older subjects with higher levels of body mass index (BMI), waist circumference, SBP, DBP, obesity, diabetes, and smoking had a high risk of high PP (*P* < 0.001).

**Table 1:** Comparison of baseline clinical characteristics among groups of normal pulse pressure and high pulse pressure

Indicators	Normal pulse pressure group (n = 438)	High pulse pressure group (n = 460)	*P* value
Sex			
Males	275	243	0.004
Females	163	217	
Age (year)	56.94 ± 13.38	65.01 ± 11.36	< 0.001
Height (cm)	164.70 ± 7.51	163.36 ± 7.92	0.008
Weight (kg)	64.93 ± 11.58	66.34 ± 11.40	0.065
BMI (kg/m^2^)	23.88 ± 3.67	24.79 ± 3.42	< 0.001
Waist circumference (cm)	84.87 ± 10.23	88.30 ± 9.66	< 0.001
SBP (mmHg)	138.05 ± 19.64	174.93 ± 23.83	< 0.001
DBP (mmHg)	87.69 ± 17.56	93.28 ± 17.31	< 0.001
PP (mmHg)	50.36 ± 8.14	81.65 ± 16.16	< 0.001
Obesity (%)	156 (35.62)	244 (53.04)	< 0.001
Diabetes (%)	103 (23.52)	198 (43.04)	< 0.001
Smoking (%)			
Smoker	275 (62.79)	324 (70.43)	< 0.001
Former-smokers	118 (26.94)	71 (15.44)	
Nonsmoker	45 (10.27)	65 (14.13)	
Drinking (%)	63 (14.38)	38 (8.26)	0.005

Data are expressed as mean and standard deviation (SD). *P* values were corrected using FDR

*BMI* body mass index, *SBP* systolic blood pressure, *DBP* diastolic blood pressure, *PP* pulse pressure, *DR* false discovery rate

### Associations between *BRD4* and *PIN1* SNPs and high PP

Of the five SNPs in *BRD4* and *PIN1* analyzed in this study, one SNP (rs2233679) was excluded because of significant deviation from HWE (*P* < 0.05), whereas the other SNPs satisfied the HWE in the control groups. Allele frequencies and basic information for all SNPs are shown in Table 2 and Additional file 1. The frequency of rs4808278 genotypes (*P* = 0.046878) was significantly different between normal PP and high PP groups. No significant difference was found in the genotype distribution of rs2233678, rs2233682, and rs2287838 between the two groups.

**Table 2:** Genotype and allele frequencies of four single nucleotide polymorphisms in the *BRD4* and *PIN1* genes in normal and high pulse pressure groups

SNPs	Genotypes and alleles	Frequencies N (%)		*P* value
		Case (n = 460)	Control (n = 438)	
	AA	250 (0.543)	240 (0.548)	0.046878
*BRD4*	AT	164 (0.357)	173 (0.395)	
rs4808278	TT	46 (0.100)	25 (0.057)	
	Allele, T (%)	256 (0.278)	223 (0.255)	0.256363
	GG	443 (0.963)	418 (0.954)	0.511810
*PIN1*	CG	17 (0.037)	20 (0.046)	
rs2233678	CC	0 (0)	0 (0)	
	Allele, C (%)	17 (0.018)	20 (0.023)	0.516271
	GG	438 (0.952)	422 (0.963)	0.400651
*PIN1*	AG	22 (0.048)	16 (0.037)	
rs2233682	AA	0 (0)	0 (0)	
	Allele, A(%)	22 (0.024)	16 (0.018)	0.405751
	CC	225 (0.489)	232 (0.530)	0.476216
*PIN1*	CT	184 (0.400)	162 (0.370)	
rs2287838	TT	51 (0.111)	44 (0.100)	
	Allele, T (%)	286 (0.311)	250 (0.285)	0.238116

A chi-squared test was used to calculate the *P* values

Three risk prediction models (additive model, dominant model, and recessive model) were used to evaluate the association between these gene polymorphisms and high PP risk. The data show that rs4808278 is markedly associated with high PP under the additive and recessive models. The adjusted *P* value was further increased after adjusting for age, weight, waist circumference, drinking, smoking, hypertension, and diabetes. As a result, carriers of the rs4808278-A allele have a higher risk of developing high PP compared those carrying the rs4808278-TT genotype after adjustment for confounding factors (OR = 0.400; 95% CI: 0.217–0.737; *P* = 0.003). No significance was observed for the remaining polymorphisms under all models (see Additional file 2: Table S2).

### Haplotype analyses of *BRD4* and *PIN1* SNPs

SHESIS software was used to perform haplotype analyses. Compared to the control group, no significant difference was found in haplotypes of the *PIN1* gene in the case group (see Additional file 2: Table S3).

### GMDR analysis of *BRD4* and *PIN1* SNPs and high PP risk

The GMDR model was used to investigate the best interaction combination among the four SNPs and smoking on high PP risk. Table 3 summarizes the results obtained from GMDR analysis for gene–gene and gene-diabetes interactions. The best three-locus models for gene-diabetes interaction (*P* = 0.0107) were rs4808278, rs2287838, and diabetes after adjusting for the covariates (age, sex, smoking, drinking and BMI), of which the cross-validation consistency was 9/10. However, we did not find a significant any-locus model among the four SNPs.

**Table 3:** GMDR analysis on the best SNP-SNP and gene-diabetes interaction models

Locus no	Best combination	Cross-validation consistency	Testing accuracy	*P* values
SNP-SNP interactions^a^				
2	rs2233682, rs4808278	8/10	0.5093	0.1719
3	rs2233678, rs2233682, rs4808278	7/10	0.4991	0.1719
Gene-diabetes interactions^b^				
2	rs4808278, diabetes	10/10	0.5783	0.0107
3	rs2233678, rs4808278, diabetes	9/10	0.5747	0.0107

*GMDR* generalized multifactor dimensionality reduction, *SNP* single nucleotide polymorphism, *BMI* body mass index

^a^Adjusted for age, sex, smoking, drinking, BMI and diabetes

^b^Adjusted for age, sex, smoking, drinking and BMI

### Mapping of associated SNPs at expression quantitative trait loci (eQTLs)

Rs4808278 was annotated using the RegulomeDB, a database that identifies DNA features and regulatory elements in non-coding regions of the human genome. Known and predicted regulatory DNA elements include DNase hypersensitivity regions, transcription factor (TF) binding sites, and promoter regions that have been biochemically characterized to regulate transcription [17]. Therefore, we investigated the function of rs4808278 in gene expression regulation by exploring the RegulomeDB. The RegulomeDB score of rs4808278 in *BRD4* was 4, suggesting that rs4808278 may be part of a TF binding site or DNase hypersensitive region.

## Discussion

Scholars are paying more attention to heredity as an important factor in high pulse differential pressure [18, 19]. To investigate a potential association between five promising polymorphisms in the *BRD4* and *PIN1* genes and high PP risk in a southeastern Chinese population, we designed this pilot case–control study that includes 666 hypertensive patients and 232 normotensive subjects according to exclusion and inclusion criteria. After excluding one SNP (rs2233679) that was not in HWE, we analyzed the remaining four SNPs. There was no statistically significant correlation between the four SNPs and hypertension risk, but we unexpectedly found that rs4808278 in *BRD4* was associated with high PP risk, suggesting that *BRD4* may play an important role in high PP progression. This discovery may provide a new therapeutic target gene.

*BRD4*, a member of the bromo and extra-terminal family, plays a role in various vascular pathologies. Knocking down *Brd4* with siRNA mitigates neointima formation and vascular remodeling [20, 21]. The protein also regulates vascular endothelial growth factor (VEGF)-induced angiogenesis by activating phosphorylation of vascular endothelial growth factor receptor 2 (VEGFR2) and P21 activated kinase 1 (PAK1) [22, 23]. To some extent, these pathologies are related to changes in vascular elasticity, which may affect PP variation. Through binding with acetylated RelA, *BRD4* enhances activation of NF-κB signaling to induce natriuretic peptide precursor A (*NPPA*) and natriuretic peptide precursor B (*NPPB*) transcription [24]. Our previous research also found that a SNP in the *NPPB* gene is associated with high PP [14]. *BRD4* plays a critical role in propagating inflammation and promoting vascular calcification in particular [7, 8]. More importantly, *BRD4*, a transcriptional regulator, mediates inflammatory transcription, atherogenic endothelial response, and atherosclerosis [10, 11]. Vascular damage, an antecedent to atherosclerosis, leads to an increase in PP and to atherosclerosis, which in turn results in large-vessel stiffening and increased wave reflection. While it is not clear what is the incipient event in this cycle, it is clear that, once initiated, a vicious cycle promoting disease progression ensues. The association analysis of PP with different genotypes show that rs4808278 genotype TT carriers have a lower risk than AT and AA genotypes carriers to have higher PP (OR = 0.405; 95% CI: 0.220–0.745; *P* = 0.004), which indicates that the T allele has a protective effect. Although rs4808278 is located in an intron of *BRD4*, the RegulomeDB score of rs4808278 in *BRD4* is 4, which suggests that rs4808278 may be part of a TF binding site or DNase hypersensitive region. This suggests that rs4808278 may affect PP by regulating *BRD4* expression. Few studies have reported on SNPs in *BRD4* and high PP. Our results may provide new research directions on high PP.

High PP, diabetes mellitus, and atherosclerosis are closely related, and PP is considered an independent predictor of new-onset diabetes [25, 26]. Therefore, diabetes and the four SNPs were included in the GMDR analysis. We found a significant three-locus model (*P* = 0.0010) involving rs4808278, rs2233678, and diabetes after adjusting the covariates. The detailed mechanism underlying this interaction is unknown, but all of them are associated with vascular elasticity, which may be the basis for high PP.

There are several limitations in the present work. Firstly, only five SNPs were studied. Secondly, this is a retrospective case–control study. Our original intention was to look for the relationship between SNPs in *BRD4*/*PIN1* and hypertension, but we did not find any association. Thirdly, the presence of atherosclerosis, an important factor causing an increase in PP, was not recorded in the patients.

## Conclusion

In conclusion, our results indicate that genetic mutations within the *BRD4* gene (rs4808278) could affect the susceptibility to high PP in a southeastern Chinese population.

## Supplementary information

**Additional file 1:** SNPs in this research.

**Additional file 2: Table S1** Description and primer sequences of the single nucleotide polymorphisms (SNPs) used for polymerase chain reaction (PCR) analysis. **Table S2** Prediction of high pulse pressure risk of four single nucleotide polymorphisms in the *BRD4* and *PIN1* genes under additive, dominant, and recessive models. **Table S3** Allele combination analysis of single nucleotide polymorphisms in the *PIN1* gene and risk prediction for high pulse pressure.

## Data Availability

The datasets generated and analysed during the current study are included within the article.

## Abbreviations

PP: Pulse pressure
SBP: Systolic blood pressure
DBP: Diastolic blood pressure
BP: Blood pressure
NO: Nitric oxide
SNP: Single nucleotide polymorphism
EDTA: Ethylene diamine tetraacetic acid
PCR-LDR: Polymerase chain reaction ligase detection reaction
SDs: Standard deviations
HWE: Hardy–Weinberg equilibrium
ORs: Odds ratios
CIs: Confidence intervals
GMDR: Generalized multifactor dimensionality reduction
FDR: False discovery rate
BMI: Body mass index
eQTLs: Expression quantitative trait loci
TF: Transcription factor
VEGF: Vascular endothelial growth factor
VEGFR2: Vascular endothelial growth factor receptor 2
PAK1: P21 activated kinase 1
*NPPA*: Natriuretic peptide precursor A
*NPPB*: Natriuretic peptide precursor B
