# Supplementary material for: BRD4 and PIN1 gene polymorphisms are associated with high pulse pressure risk in a southeastern Chinese population
Source: BMC Cardiovasc Disord. 2020 Nov 4;20:475. doi: 10.1186/s12872-020-01757-x (PMC7640679; doi:10.1186/s12872-020-01757-x)
Supplement: Supplementary file 2 — Additional file 2: Table S1 Description and primer sequences of the single nucleotide polymorphisms (SNPs) used for polymerase chain reaction (PCR) analysis. Table S2 Prediction of high pulse pressure risk of four single nucleotide polymorphisms in the BRD4 and PIN1 genes under additive, dominant, and recessive models. Table S3 Allele combination analysis of single nucleotide polymorphisms in the PIN1 gene and risk prediction for high pulse pressure. [file 12872_2020_1757_MOESM2_ESM.docx]

**Table S1** Description and primer sequences of the single nucleotide polymorphisms (SNPs) used for polymerase chain reaction (PCR) analysis.

| **Gene** | **SNP ID** | **Functional consequence** | **Allele Change** | **Primer sequences** |
| --- | --- | --- | --- | --- |
| ***BRD4*** | **rs4808278** | intron variant | A>T | 5′-TGAAACCCACACCACTTCAGACGAC[A/T]ACAGCTAAGGAAGCAAAAAACTTGG-3′ |
| ***PIN1*** | **rs2233678** | nc transcript variant,upstream variant 2KB | G>C | 5′-ACTCTATTTTAAGTTGGCTAAAACT[A/C/G]AGCCCATCTCCTCCCACCTGCACCT-3′ |
|  | **rs2233679** | nc transcript variant,upstream variant 2KB | C>T | 5′-TCCAGAGCCTAGGGAAAAGTTGAGC[C/T]CTGCAAAAAATAAAAGAGGCTGGGT-3′ |
|  | **rs2233682** | nc transcript variant,synonymous codon | G>A | 5′-TCAACCACATCACTAACGCCAGCCA[A/G]TGGGAGCGGCCCAGCGGCAACAGCA-3′ |
|  | **rs2287838** | intron variant | C>T | 5′-TAGAGATGATGCCAGGAAGAAAGTG[C/T]TGCAGCCCCCCTCCCTGTGTGGCCC5′- |

^*^SNP: single nucleotide polymorphism; PIN1: peptidylprolyl cis/trans isomerase, NIMA-interacting 1; BRD4: bromodomain containing 4.

**Table S2** Prediction of high pulse pressure risk of four single nucleotide polymorphisms in the *BRD4* and *PIN1* genes under additive, dominant, and recessive models.

| **Polymorphisms** |  | **OR;95%CI;*P* value** | **OR;95%CI;*P** value** |
| --- | --- | --- | --- |
| **Additive model** |  |  |  |
| **rs4808278** | AA | 0.566, 0.337-0.951,0.031 | 0.410,0.219-0.766,0.005 |
|  | AT | 0.515, 0.303-0.877,0.015 | 0.385,0.202-0.732,0.004 |
|  | TT | Reference | Reference |
| **rs2233678** | GG | 1.247,0.644-2.413,0.513 | 0.873,0.402-1.895,0.730 |
|  | CG | Reference | Reference |
|  | CC | NA | NA |
| **rs2233682** | GG | 0.755,0.391-1.457,0.402 | Reference |
|  | AG | Reference | 0.737,0.342-1.591,0.437 |
|  | AA | NA | NA |
| **rs2287838** | CC | 0.837,0.537-1.303,0.430 | 0.873,0.520-1.465,0.607 |
|  | CT | 0.980,0.622-1.545,0.930 | 1.045,0.614-1.778,0.871 |
|  | TT | Reference | Reference |
| **Dominant model** |  |  |  |
| **rs4808278** | AA | 0.982, 0.755-1.277, 0.893 | 0.911,0.670-1.238,0.552 |
|  | AA+AT | Reference | Reference |
| **rs2233678** | GG | 1.247,0.644-2.413,0.513 | 0.873,0.402-1.895,0.730 |
|  | CG+CC | Reference | Reference |
| **rs2233682** | GG | 0.755,0.391-1.457,0.402 | 0.737,0.342-1.591,0.437 |
|  | AG+AA | Reference | Reference |
| **rs2287838** | CC | 0.850,0.654-1.105,0.225 | 0.843,0.622-1.144,0.273 |
|  | CT+TT | Reference | Reference |
| **Recessive model** |  |  |  |
| **rs4808278** | AA+AT | 0.545,0.329-0.903,0.019 | 0.400,0.217-0.737,0.003 |
|  | TT | Reference | Reference |
| **rs2233678** | GG+CG | NA | NA |
|  | CC | Reference | Reference |
| **rs2233682** | GG+AG | NA | NA |
|  | AA | Reference | Reference |
| **rs****2287838** | CC+CT | 0.896,0.585-1.372,0.612 | 0.944,0.574-1.554,0.821 |
|  | TT | Reference | Reference |

OR: odds ratio; 95 % CI: 95 % confidence interval. P* values were adjusted for age, weight, waist circumference, drinking, smoking, hypertension, and diabetes. NA: Because the number of patients with this genotype was zero, the risk could not be calculated.

**Table S3** Allele combination analysis of single nucleotide polymorphisms in the *PIN1* gene and risk prediction for high pulse pressure.

|  | case | control | OR [95%CI] | P |
| --- | --- | --- | --- | --- |
| *PIN1* gene (rs2233678-rs2233682-rs2287838) | |  |  |  |
| C-A-C | 0.00(0.000) | 0.62(0.001) | Reference group | - |
| G-G-C | 603.03(0.655) | 594.92(0.679) | 0.896 [0.729~1.100] | 0.294866 |
| G-G-T | 277.97(0.302) | 245.70(0.280) | 1.116 [0.909~1.371] | 0.294866 |

Abbreviations: 95 % CI: 95 % confidence interval; OR: odds ratio.
